# Casein Supplementation Timing and Exercise Performance in Soccer Players: Pre-Sleep vs. Post-Exercise Intake—A Randomized Controlled Trial

**DOI:** 10.3390/nu17243938

**Published:** 2025-12-17

**Authors:** Serdar Bayrakdaroğlu, Zeynep Hazal Ateş, Halil İbrahim Ceylan, Murat Kul, Raul Ioan Muntean, Wissem Dhahbi

**Affiliations:** 1 Faculty of Sports Sciences, Gümüşhane University, Gumushane 29100, Türkiye; bayrakdaroglu85@gmail.com (S.B.); bakhazalcim@hotmail.com (Z.H.A.); 2 Faculty of Sports Sciences, Atatürk University, Erzurum 25100, Türkiye; 3 Faculty of Sports Sciences, Bayburt University, Bayburt 69000, Türkiye; muratkul@bayburt.edu.tr; 4 Faculty of Law and Social Sciences, University “1 Decembrie 1918” of Alba Iulia, 510009 Alba Iulia, Romania; 5 Research Unit: Sport Sciences, Health and Movement, High Institute of Sport and Physical Education of Kef, University of Jendouba, Kef 7100, Tunisia; wisem.dhahbi@gmail.com; 6 Training Department, Police College, Qatar Police Academy, Doha 7157, Qatar

**Keywords:** casein, timing, anaerobic performance, recovery, soccer players

## Abstract

**Background:** Nutrient timing strategies may influence recovery and performance in athletes; however, the effects of ingesting casein protein before sleep versus immediately after exercise remain unclear. This study compared the acute effects of pre-sleep and post-exercise ingestion of casein on high-intensity anaerobic performance in highly trained soccer players. **Methods:** Twenty-four highly trained male soccer players (mean age: 20.6 ± 1.8 years) were randomly assigned to three groups: post-exercise casein ingestion group (PECIG; *n* = 8), pre-sleep casein ingestion group (PSCIG; *n* = 8), and control group (CG; *n* = 8). Following a standardized high-intensity resistance training protocol, participants consumed 30 g of micellar casein dissolved in 300 mL of water either immediately after exercise or 30–60 min before bedtime. Performance was assessed using the Countermovement Jump (CMJ), the Illinois Agility Test (IAT), and the Running-based Anaerobic Sprint Test (RAST), which were conducted both before and 24 h after the training session. **Results:** Repeated-measures ANOVA revealed significant group-by-time effects for CMJ (F = 8.21, *p* = 0.002, η^2^p = 0.36) and RAST performance variables, including peak power (F = 6.94, *p* = 0.003, η^2^p = 0.32), mean power (F = 7.42, *p* = 0.002, η^2^p = 0.34), and fatigue index (F = 5.87, *p* = 0.008, η^2^p = 0.28). Post hoc analyses showed that PSCIG significantly improved CMJ (Δ = +1.45 cm; *p* < 0.001, d = 2.04) and RAST mean power (Δ = +125.14 W; *p* = 0.002, d = 1.55) compared with the control condition. PECIG significantly enhanced RAST peak power (Δ = +205.79 W; *p* = 0.001, d = 1.64), mean power (Δ = +128.43 W; *p* = 0.013, d = 1.24), and fatigue index (Δ = +4.12 W/s; *p* = 0.010, d = 1.29) relative to CG. No performance differences were observed between PSCIG and PECIG timing conditions (all *p* > 0.05). **Conclusions:** Both pre-sleep and post-exercise casein ingestion enhanced anaerobic performance in highly trained soccer players, with each timing method favoring different performance outcomes. However, neither strategy demonstrated superiority over the other, suggesting that casein timing may be flexibly applied according to athletes’ preferences and training contexts.

## 1. Introduction

Soccer imposes substantial anaerobic demands through repeated high-intensity actions, including sprinting, jumping, and directional changes, performed over 90 min [1,2]. These explosive movements induce muscle damage and metabolic stress, impairing force-generating capacity and requiring effective recovery strategies [3,4]. Nutritional strategies represent accessible and effective recovery interventions [5,6]. Timely protein intake promotes muscle repair and anabolic processes [7], whereas inadequate intake impairs recovery and performance [8].

Among dietary proteins, casein presents distinct advantages for recovery applications. Unlike fast-digesting proteins such as whey, casein forms gastric curds that delay absorption, yielding sustained aminoacidemia for 7–8 h post-ingestion [9]. This slow-release profile provides prolonged anabolic stimulus, particularly during extended fasting periods such as overnight sleep [10]. Casein supplies all essential amino acids, including 3.4 g of leucine per 30 g serving, sufficient to stimulate muscle protein synthesis [9]. Compared with plant-based proteins (soy, pea), casein exhibits superior digestibility (95% vs. 85–90%) and a higher leucine content, both of which enhance anabolic potency [11]. These properties position casein as an optimal protein source for investigating timing-dependent recovery mechanisms.

The nutrient timing hypothesis posits that temporal manipulation of macronutrient delivery modulates the magnitude and duration of anabolic signaling cascades [12]. This temporal sensitivity derives from exercise-induced perturbations in muscle protein turnover, insulin sensitivity, and amino acid transporter expression [7]. Protein ingestion timing exploits these metabolic windows through distinct mechanisms. Post-exercise ingestion leverages exercise-induced increases in muscle perfusion and mechanistic target of rapamycin complex 1 (mTORC1) sensitivity, thereby maximizing acute anabolic responses during the 0–2 h window, when muscle protein synthesis rates are elevated 2–3-fold above baseline [13]. Pre-sleep ingestion targets the prolonged overnight post-absorptive phase (7–8 h), during which amino acid availability would otherwise decline, attenuating the nocturnal suppression of muscle protein synthesis [10,14]. Casein’s delayed gastric emptying and micellar structure produce sustained plasma amino acid elevations (peak at 3–4 h, elevated through 7–8 h), contrasting with whey protein’s rapid peak (1 h) and return to baseline (3–4 h) [9]. This pharmacokinetic profile aligns casein supplementation with extended fasting periods, during which prolonged aminoacidemia sustains anabolic processes, whereas fast proteins optimize acute post-exercise windows.

Exercise simultaneously increases both muscle protein breakdown and protein synthesis [15]. To shift this balance toward anabolism during post-exercise recovery, one of the most effective nutritional strategies is to increase protein intake [7]. Proteins provide the essential building blocks for repairing and remodeling muscle tissue, thereby supporting the continuity of anabolic processes. In addition, they contribute to RNA and DNA synthesis, facilitate the formation of connective tissues, and enhance immune function, ultimately accelerating the recovery process [16]. Conversely, inadequate protein intake after exercise may suppress muscle protein synthesis and accelerate catabolic processes, impairing recovery and potentially reducing performance [17].

During periods of intense training or under conditions of energy restriction, maintaining a daily protein intake of 1.4–2.0 g/kg/day is essential for preserving lean body mass [18]. However, excessive protein intake does not increase muscle mass; instead, it elevates amino acid oxidation and nitrogen excretion, thereby limiting net protein balance [18,19]. Furthermore, short-term fluctuations in protein intake can negatively impact the anabolic response. In particular, among athletes accustomed to a high-protein diet, sudden reductions in protein intake may impair post-exercise anabolic processes and lead to inaccurate estimates of protein requirements based on oxidation-based measurements [20]. Therefore, not only the total quantity of protein but also its quality and timing are critical determinants for optimizing sports nutrition strategies.

Protein intake among soccer players is generally low, and their diets often do not differ significantly from those of the general population. One of the most effective strategies to compensate for insufficient protein intake is the use of dietary protein supplements following exercise [21]. In a study of soccer players, Poulios et al. [22] reported that higher protein intake was associated with faster recovery of muscle strength, sprint, and jump performance, and with a more rapid return of oxidative stress markers to baseline levels. Among commonly used dietary protein supplements, casein (CAS) is particularly noteworthy due to its slow digestion rate and potential to optimize muscle repair when consumed either post-exercise [23] or before sleep [24].

Casein, the primary protein component of milk, provides a complete amino acid profile, including essential amino acids, and thus represents an effective source for meeting protein requirements [9]. Its nutritional impact is further distinguished by its slow-digesting properties. By prolonging amino acid release in the digestive tract, casein provides sustained amino acid availability, particularly throughout the night, thereby supporting muscle protein synthesis and earning recognition as a “slow-release” protein source [10]. For this reason, it is often consumed during extended periods of fasting or before sleep. Depending on specific circumstances, a daily intake of approximately 30–40 g of casein (typically post-exercise or pre-sleep) is recommended to optimize recovery [18]. Casein timing effects demonstrate population-specific variability. In resistance-trained individuals, pre-sleep casein ingestion (30–40 g) enhances overnight myofibrillar protein synthesis rates and augments strength gains during training interventions [10,14]. However, team-sport athletes present distinct physiological demands characterized by repeated high-intensity efforts rather than maximal force production. Emerging evidence in soccer players indicates that pre-sleep casein (40 g) accelerates functional recovery, improving countermovement jump performance and reactive strength index 24 h post-match compared with placebo [25]. Similarly, pre-sleep casein combined with probiotics enhanced anaerobic power output and reduced fatigue indices in soccer players following high-intensity exercise [26]. Conversely, post-exercise protein ingestion (milk protein concentrate, 80% casein) expedited recovery of sprint performance and knee extensor strength during congested match schedules [22]. These team-sport findings suggest timing-dependent effects on functional performance outcomes, yet direct comparisons between pre-sleep and post-exercise casein ingestion remain absent in soccer populations, limiting evidence-based timing recommendations for practitioners.

The present study addressed this gap through a randomized controlled trial in highly trained soccer players. The primary research question was: Does timing of casein supplementation (immediate post-exercise vs. 30–60 min pre-sleep) differentially influence 24 h recovery of anaerobic performance following high-intensity resistance exercise? We tested two competing hypotheses: (1) pre-sleep casein ingestion would enhance recovery through sustained overnight muscle protein synthesis, yielding superior performance restoration in explosive power metrics (countermovement jump height, anaerobic power output); (2) post-exercise casein ingestion would optimize acute recovery processes, yielding superior fatigue resistance and peak power preservation through immediate amino acid availability during heightened muscle sensitivity. Secondary outcomes included agility performance and inter-group comparisons to determine whether one timing strategy demonstrates superiority across multiple performance domains.

## 2. Methods

### 2.1. Participants

A priori sample size estimation was conducted using G*Power 3.1.9.7 software (Heinrich-Heine-Universität Düsseldorf, Düsseldorf, Germany) following established recommendations for strength and conditioning research [27,28]. Power analysis was based on anticipated medium-to-large effect sizes (Cohen’s d = 0.8) derived from previous casein supplementation investigations in athletic populations, particularly pre-sleep protein interventions that have demonstrated meaningful performance enhancements [29]. With α = 0.05, statistical power = 0.80, and a three-group design, the analysis indicated a minimum sample size of *n* = 21 participants (7 per group) to detect statistically significant between-group differences. To account for potential attrition and ensure adequate statistical power to detect meaningful performance changes, the final sample comprised *n* = 24 participants (8 per group), providing >85% statistical power for the primary outcome comparisons and enhancing precision for effect-size estimation across secondary performance parameters.

A total of 30 male soccer players were screened for eligibility. Participants were recruited from highly trained, semi-professional soccer players actively competing at comparable levels. Goalkeepers were excluded to avoid positional bias. Two players declined participation, and four did not meet the inclusion criteria, leaving 24 players who completed the study (mean ± SD, age 20.62 ± 1.90 years; height 173.5 ± 5.05 cm; body mass 70.79 ± 5.92 kg; BMI 23.65 ± 2.03 kg/m^2^; body fat percentage 15.00 ± 5.28%). Participants were classified as Tier 3 athletes according to the framework proposed by McKay et al. [30], corresponding to highly trained individuals competing at national or international junior/development levels with structured training programs exceeding 6 h per week. To be eligible, players were required to be between 18 and 25 years of age, to have at least three years of competitive soccer experience, to have engaged in structured training at least three times per week during the previous six months, and to obtain clearance on the Physical Activity Readiness Questionnaire (PAR-Q) [31,32]. Players were excluded if they had suffered any musculoskeletal injury or undergone surgery within the past six months, if they reported milk or casein intolerance or allergy, or if they had used ergogenic supplements such as protein or creatine regularly during the previous four weeks. The study protocol was conducted in accordance with the ethical standards of the Declaration of Helsinki (World Medical Association, 2013) and was approved by the Gümüşhane University Scientific Research and Publication Ethics Committee (Approval No: 2024/8, 25 October 2024); NCT Number: NCT07170228. All participants were fully informed about the study’s purpose, scope, and methodology and provided written informed consent before participation.

### 2.2. Experimental Procedures

**Figure 1:**
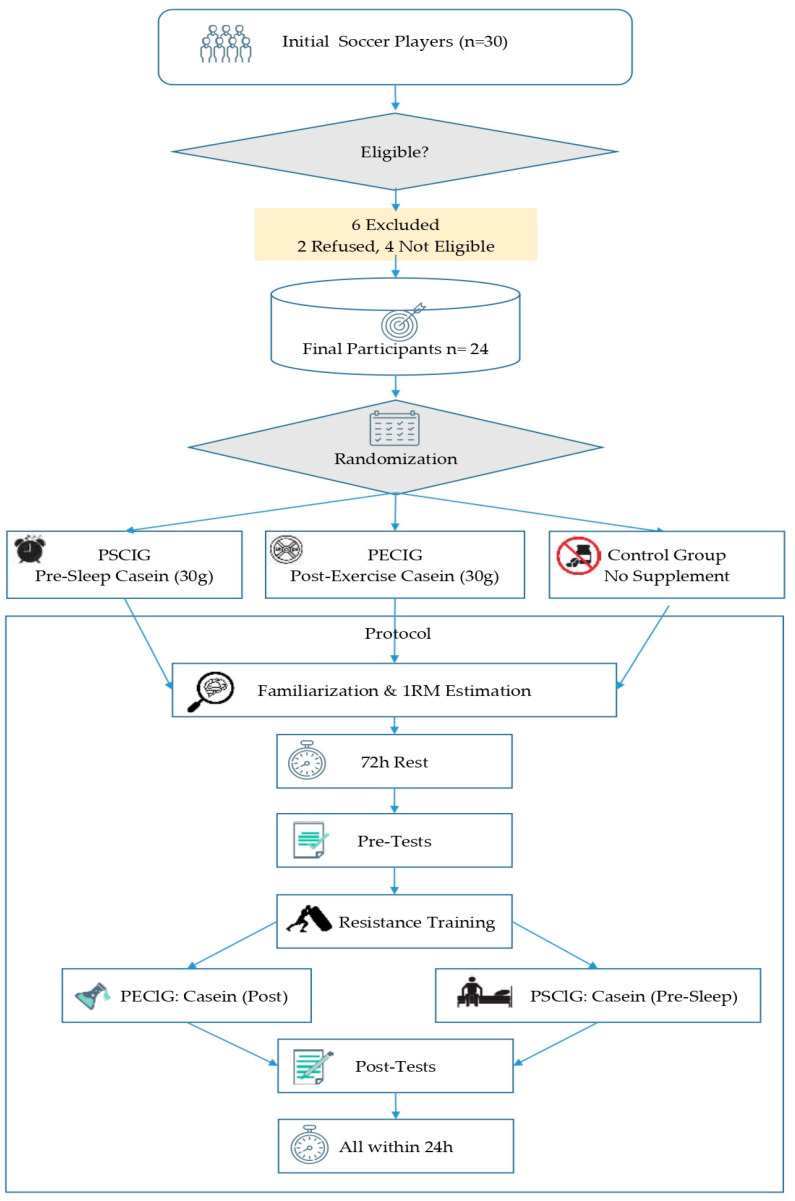
Experimental Procedures.

This study employed a randomized controlled experimental design to evaluate the effects of the timing of pre-sleep and post-exercise casein supplementation on post-exercise recovery. Participants were allocated into three groups: pre-sleep casein ingestion group (PSCIG; *n* = 8), post-exercise casein ingestion group (PECIG; *n* = 8), and control group (CG; *n* = 8). To ensure balanced allocation, players were stratified by playing position (defender, midfielder, forward) and randomized using a stratified block randomization procedure. Randomization was performed using a computer-generated sequence and concealed allocation www.randomizer.org (accessed on 6 October 2025). All participants completed the familiarization session, pre-test assessments, the standardized high-intensity resistance training protocol, and post-test assessments under controlled environmental conditions (20–24 °C ambient temperature). Seventy-two hours before the pre-tests, participants attended a familiarization session to introduce the measurement protocols, including the countermovement jump (CMJ), the Illinois Agility Test, and the Running-based Anaerobic Sprint Test (RAST). During this session, the procedures were explained and demonstrated, and all participants practiced the standardized execution of each test to minimize potential learning effects. On the same day, one-repetition maximum (1RM) tests were performed in the back squat and bench press to determine participants’ individual maximal strength levels. A standard 1RM testing protocol outlined by the National Strength and Conditioning Association (NSCA) [33] was applied. The 1RM test was performed on a Smith machine for both bench press (BP) and back squat (BS), as previously described by Ritti-Dias et al. [34]. The testing procedure was adapted from the protocol proposed by Macht et al. [35]. The obtained 1RM values were subsequently used to determine the training loads for the standardized high-intensity resistance training protocol. Seventy-two hours after the familiarization session, participants underwent the pre-test assessments. The following day, all participants performed the standardized high-intensity resistance training protocol under the supervision of certified trainers at a fitness facility. Ten to fifteen minutes after completing the exercise, the PECIG consumed 30 g of micellar casein (Nutrend^®^ Micellar Casein, Nutrend D.S., Olomouc, Czech Republic) dissolved in 300 mL of water [36]. In contrast, the PSCIG was administered at the same dose 30–60 min before bedtime [29]. Pre-sleep intake was additionally verified through direct monitoring. The CG received no supplementation. All supplements were prepared and provided by the research team to ensure dosage accuracy and compliance. Exactly 24 ± 1 h after resistance training, all participants completed the post-tests in the same order and using the same protocols as in the pre-tests. All participants completed the study protocol, and no data loss occurred. The assessors conducting the measurements were blinded to group allocation. To minimize potential circadian rhythm influences, all protocols were performed at the same time of day (14:00–16:00) [37]. Participants were instructed to abstain from alcohol and caffeine for 24 h before testing and to maintain their habitual sleep patterns. Additionally, dietary intake was monitored using food logs and standardized throughout the study. The study procedure is illustrated in Figure 1.

### 2.3. Diet Standardization

Throughout the study period, all participants followed a standardized soccer-specific diet designed and supervised by a dietitian on the research team. In soccer nutrition, it is generally recommended that players consume 40–60 kcal/kg body weight per day (approximately 3500–4000 kcal/day) on days with low training intensity. Macronutrient distribution should include 3–8 g/kg/day of carbohydrates, 1.2–2.0 g/kg/day of protein, and fats providing 25–30% of total energy intake [38,39,40,41,42]. To ensure dietary compliance, participants were provided with 24-h dietary record forms and instructed to record all foods and beverages consumed accurately. Participants estimated portion sizes using a validated photographic food atlas (Turkish Food Photo Album) [43,44] supplemented with household measures (cups, spoons, standard serving sizes). To promote adherence, daily reminder messages were sent, and compliance was monitored through online communication channels. All 24-h dietary records were analyzed using validated nutrition analysis software (BeBIS 11, Nutrition Information System, Istanbul, Turkey), which automatically calculated total energy intake and macronutrient composition based on the Turkish Food Composition Database. For each participant, three nonconsecutive dietary records (two weekdays and one weekend day) were collected over 7 days, and the mean values for total energy intake and macronutrient distribution are presented in Table 1.

### 2.4. Warm-Up Protocol

The warm-up protocol was conducted separately for the pre- and post-tests and for the resistance training session. Before the pre- and post-tests, all participants performed a standardized dynamic warm-up lasting approximately 10 min. The protocol began with 3 min of light running at a pace of 8–10 km/h, followed by 5–7 min of dynamic mobility drills, including high knees, butt kicks, lateral shuffles, carioca runs, and walking lunges, in addition to two 10 m acceleration sprints [45]. Before the resistance training session, participants completed a warm-up lasting approximately 10–15 min. This consisted of 3 min of cycling on an ergometer at a light pace (8–10 km/h), followed by dynamic mobility exercises (walking lunges, arm circles, hip circles, inchworm walkouts, and side lunges) and activation drills (glute bridges, band pull-aparts, and plank shoulder taps). Finally, a specific warm-up was performed for the main lifts, consisting of two sets of 8 repetitions each of back squat and bench press at 40% of the estimated 1RM [45].

### 2.5. Standardized High-Intensity Resistance Training Protocol

To provide participants with a homogeneous and reproducible physiological stressor, a standardized high-intensity resistance training protocol targeting both upper- and lower-body muscle groups was implemented. The protocol was designed to induce muscle damage and fatigue. The main exercises, back squat and bench press, were performed at 75% of participants’ previously determined 1RM values, with three sets of 8–12 repetitions. For the auxiliary exercises—Romanian deadlift, bent-over row, walking lunge, and seated shoulder press—10-repetition maximum (10RM) loads were determined and performed using the same set-repetition scheme. The 10RM test is a widely used and reliable method for determining training loads in resistance exercise [33,46]. This approach is also consistent with the resistance training literature, which recommends an 8–12 repetition range as an effective strategy for load prescription and training progression aimed at muscular hypertrophy [47]. Rest intervals were standardized as 90 s between sets and 120 s between exercises [48,49]. Training intensity was monitored using the resistance exercise–specific 0–10 Rating of Perceived Exertion (OMNI-RES) scale, with all sets performed at an RPE of 8–9. All movements were executed with a controlled tempo (eccentric–pause–concentric: 3–0–1) [50,51,52]. The main training session, excluding the warm-up, lasted approximately 40–45 min., while the total session, including the warm-up, lasted about 50–60 min.

### 2.6. Data Collection

#### 2.6.1. Anthropometric Measurements

Participants’ standing height was measured using an electronic stadiometer with a precision of 0.001 m (Seca-769, Seca Corporation, Hamburg, Germany). Body mass and body fat percentage were assessed with a bioelectrical impedance analyzer (InBody 720, Biospace, Seoul, Republic of Korea) with an accuracy of 0.01 kg [53]. All measurements were conducted by a single experienced examiner, with participants standing barefoot and wearing light sports attire (shorts and a T-shirt), in accordance with the manufacturer’s standardized protocols. Based on the obtained data, body mass index (BMI) was calculated as body mass (kg) divided by height squared (m^2^).

#### 2.6.2. Countermovement Jump (CMJ)

The CMJ performance was assessed using the Desmotec E-Board (Desmotec s.r.l., Biella, Italy), a portable evaluation platform equipped with motion analysis and real-time feedback capabilities. Following a standardized warm-up protocol, participants performed CMJs on the platform with hands fixed at the waist. During each trial, adherence to the protocol was ensured by monitoring to prevent excessive forward, backward, or lateral displacement, avoiding knee flexion during the flight phase, and maintaining proper body alignment. Each participant completed two attempts, and the highest jump height (cm) was used for analysis. A 60 s passive rest interval was provided between trials. To enhance test consistency, participants wore standardized athletic footwear during both pre- and post-test sessions. The CMJ protocol implemented with this portable device is consistent with established field assessment methods for lower-limb explosive power and neuromuscular performance, and previous large-sample investigations have supported its validity and reliability [54].

#### 2.6.3. Illinois Agility Test (IAT)

The IAT was administered during a standardized course to assess participants’ change-of-direction ability and speed. The testing area was set at 10 m in length and 5 m in width, with the start line, finish line, and turning points delineated by four marker cones. Additionally, four cones were positioned along the central line at 3.3 m intervals. Participants began the test prone on the starting line, with the chin in contact with the floor and the hands aligned with the shoulders, and initiated it at their own pace. During the trial, participants were required to sprint along the designated route, perform zigzag maneuvers between the central cones, and complete all directional changes without making contact with the markers [55,56,57]. Performance times were recorded in seconds using a wireless photocell timing system (Witty, Microgate, Bolzano, Italy) positioned at the start and finish lines. Each participant performed two trials, and the best performance time was used for subsequent analysis. Previous research has demonstrated the reliability and validity of the IAT in evaluating agility performance in male team-sport athletes [58].

#### 2.6.4. Running-Based Anaerobic Sprint Test (RAST)

Following completion of the IAT, participants were provided with a 5 min passive rest interval to ensure adequate physiological recovery and to prevent potential performance decrements in the subsequent RAST [59]. The RAST was conducted outdoors on natural grass, with players wearing soccer cleats, in accordance with protocols previously shown to be highly reliable for soccer athletes (ICC = 0.88–0.96) and suitable for field-based applications [60]. The protocol consisted of six maximal 35 m sprints, each separated by a 10 s passive recovery period. Sprint times were recorded to the nearest 0.01 s using a dual wireless photocell timing system (Witty, Microgate, Bolzano, Italy). Power output for each sprint was expressed in Watts (W) and calculated by multiplying body mass (kg) by the square of the sprint distance (35 m), then dividing this value by the sprint time (s) cubed. Peak power was defined as the highest recorded value across the six sprints, minimum power as the lowest, and mean power as the average of the power values across all six sprints. The fatigue index was obtained by subtracting the minimum power from the peak power and dividing the result by the total sprint time of the six runs [59,60,61].

### 2.7. Statistical Analysis

Statistical analyses were conducted using IBM SPSS Statistics version 29.0 (IBM Corporation, Armonk, NY, USA) and R version 4.3.1 (R Core Team, Vienna, Austria), with reproducibility ensured by fixed random-seed initialization (set.seed = 12345). Data screening encompassed completeness assessment, Shapiro–Wilk normality testing, Levene’s homoscedasticity testing, and outlier detection using standardized residuals and Cook’s distance. Distributional assumptions were corroborated through quantile-quantile plots and histogram inspection.

Primary analyses employed mixed-effects analysis of covariance (ANCOVA) with post-intervention scores as the dependent variable, group allocation as a fixed effect, pre-intervention values as covariates, and playing position as a stratification factor. Planned orthogonal contrasts examined between-group differences with family-wise error rate control via Bonferroni correction (α = 0.0167); secondary analyses utilized paired-sample t-tests for within-group changes and independent-samples t-tests for between-group change score comparisons.

Effect size quantification employed Cohen’s d with bias correction (Hedges’ g) and standardized response means (SRM) for within-group magnitudes. Confidence intervals (95% CI) were calculated using bias-corrected accelerated bootstrap procedures (B = 2000 replications). Clinical significance was assessed through the smallest worthwhile change criteria (SWC = 0.2 × between-subject SD). Statistical significance was defined as *p* < 0.05 for secondary analyses and *p* < 0.0167 for primary comparisons after multiple-comparison adjustments.

## 3. Results

### 3.1. Participant Flow and Baseline Characteristics

Twenty-four highly trained male semi-professional soccer players completed the study protocol without attrition (mean age: 20.6 ± 1.8 years; body mass: 70.8 ± 5.9 kg; height: 173.5 ± 5.1 cm; BMI: 23.7 ± 2.0 kg/m^2^; body fat: 15.0 ± 5.3%). Participants were equally distributed across three groups: CG (*n* = 8), PECIG (*n* = 8), and PSCIG (*n* = 8). Baseline demographic characteristics demonstrated adequate balance between groups, with no clinically meaningful differences observed in age (CG: 21.4 ± 2.0 years; PECIG: 20.1 ± 1.8 years; PSCIG: 20.4 ± 1.5 years), anthropometric measures, or body composition indices (Table 2).

### 3.2. Primary Outcomes: Between-Group Comparisons

Analysis of change scores revealed significant between-group differences in multiple performance parameters, corrected for multiple comparisons (α = 0.0167). Pre-sleep casein supplementation demonstrated superior efficacy compared to control conditions for CMJ performance (mean difference: 1.45 cm, 95% CI: 0.75 to 2.15 cm; Cohen’s d = 2.037; t_14_ = 4.074; *p* < 0.001) and RAST mean power output (mean difference: 125.14 W, 95% CI: 42.86 to 207.42 W; Cohen’s d = 1.552; t_14_ = 3.104; *p* = 0.002). Post-exercise casein supplementation yielded significant improvements relative to control for RAST peak power (mean difference: 205.79 W, 95% CI: 79.42 to 332.16 W; Cohen’s d = 1.640; t_14_ = 3.281; *p* = 0.001), mean power (mean difference: 128.43 W, 95% CI: 24.38 to 232.48 W; Cohen’s d = 1.239; t_14_ = 2.479; *p* = 0.013), and fatigue index (mean difference: 4.12 W/s, 95% CI: 0.87 to 7.37 W/s; Cohen’s d = 1.291; t_14_ = 2.582; *p* = 0.010). Complete pre-intervention, post-intervention, and change score data for all performance outcomes are presented in Table 3.

Detailed statistical comparisons, including within-group changes and between-group effect sizes across all measured parameters, are provided in Table 4.

**Figure 2:**
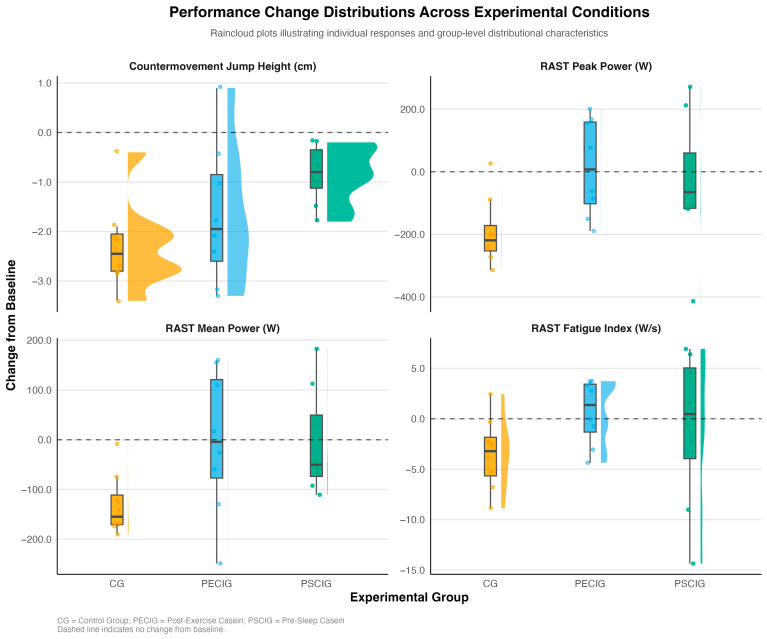
Performance changes by group and outcome. Raincloud plots display the distribution of change scores (post-intervention minus pre-intervention) for primary performance outcomes across the three experimental groups. Individual data points represent participant-level responses; box plots show the median and interquartile ranges; and half-violin plots illustrate the probability density distribution. Horizontal dashed lines indicate no change from baseline. CG = Control Group; PECIG = Post-Exercise Casein Ingestion Group; PSCIG = Pre-Sleep Casein Ingestion Group.

Visual inspection of change score distributions (Figure 2) reveals distinct response patterns across experimental conditions. The CG exhibited consistent performance decrements across all outcomes, as evidenced by negative change scores and a narrow distribution. In contrast, both intervention groups demonstrated attenuated declines or positive changes, with PSCIG showing tighter clustering around median values for CMJ height and RAST mean power. In comparison, PECIG exhibited greater variability but preserved peak power output more effectively.

The IAT time performance approached statistical significance for PSCIG versus control CG (mean difference: 0.66 s, Cohen’s d = 1.118; t_14_ = 2.235; *p* = 0.025), though this did not survive Bonferroni correction. Direct comparisons between intervention groups (PSCIG vs. PECIG) revealed no statistically significant differences across all measured parameters (*p* > 0.05 for all).

### 3.3. Secondary Outcomes: Within-Group Changes

Paired-sample analyses demonstrated significant performance decrements in the CG across all measured variables: CMJ performance (Δ = −2.29 ± 0.84 cm; standardized response mean [SRM] = −2.713; t_7_ = −7.673; *p* < 0.001), IAT time (Δ = −0.63 ± 0.51 s; SRM = −1.234; t_7_ = −3.492; *p* < 0.001), RAST peak power (Δ = −191.71 ± 103.14 W; SRM = −1.859; t_7_ = −5.257; *p* < 0.001), mean power (Δ = −131.18 ± 57.91 W; SRM = −2.265; t_7_ = −6.406; *p* < 0.001), minimum power (Δ = −93.07 ± 50.91 W; SRM = −1.828; t_7_ = −5.171; *p* < 0.001), and fatigue index (Δ = −3.45 ± 3.37 W/s; SRM = −1.024; t_7_ = −2.896; *p* = 0.004).

Both casein supplementation groups demonstrated significant within-group improvements in CMJ performance: PECIG (Δ = −1.66 ± 1.34 cm; SRM = −1.239; t_7_ = −3.505; *p* < 0.001) and PSCIG (Δ = −0.84 ± 0.55 cm; SRM = −1.523; t_7_ = −4.308; *p* < 0.001). No other within-group changes were statistically significant in the intervention groups (all *p* > 0.05).

### 3.4. Effect Size Magnification and Clinical Significance

**Figure 3:**
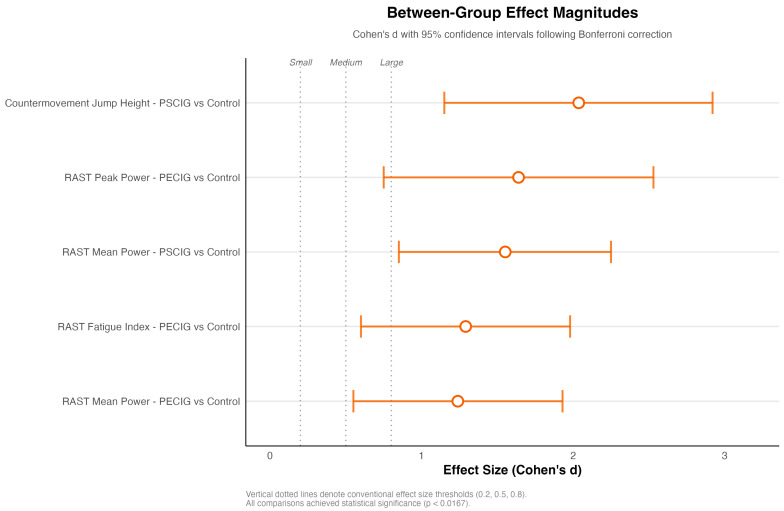
Between-group effect sizes for statistically significant comparisons. Forest plot displaying Cohen’s d effect sizes with approximate 95% confidence intervals for comparisons achieving statistical significance after Bonferroni correction (α = 0.0167). Vertical reference lines denote conventional effect size thresholds: small (d = 0.2), medium (d = 0.5), and large (d = 0.8). All displayed comparisons showed large effect sizes that exceeded clinical significance criteria.

Between-group effect sizes exceeded conventional thresholds for enormous practical significance (Cohen’s d > 0.8) in multiple domains. PSCIG supplementation yielded the largest effect for CMJ performance (d = 2.037), while PECIG supplementation demonstrated superior effects for anaerobic power parameters (peak power: d = 1.640; fatigue resistance: d = 1.291). The magnitude of the observed effects substantially exceeded the criteria for a meaningful change, indicating a performance improvement beyond measurement error. Figure 3 illustrates the effect sizes for statistically significant comparisons after Bonferroni correction, with all displayed contrasts exceeding the large effect threshold (d = 0.8) and several exceeding the very large effect threshold (d > 1.2). Notably, PSCIG versus control comparisons for CMJ performance (d = 2.037) and RAST mean power (d = 1.552) demonstrated the most pronounced ergogenic effects.

**Figure 4:**
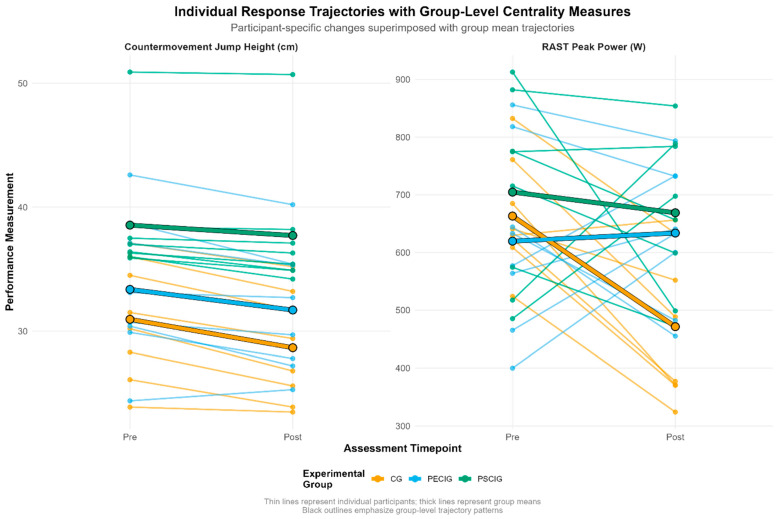
Individual and group mean changes for key outcomes. Spaghetti plots illustrate individual participant trajectories (thin lines) and group means (thick lines) from pre-intervention to post-intervention for countermovement jump height and RAST peak power. These outcomes demonstrated the most pronounced between-group differences, with distinct intervention-specific response patterns evident across supplementation timing strategies.

Individual participant response trajectories (Figure 4) reveal heterogeneous adaptation patterns across experimental conditions. For CMJ performance, the CG exhibited uniformly negative trajectories with minimal inter-individual variation. In contrast, both intervention groups demonstrated response heterogeneity, with PSCIG participants clustering more closely around the group mean trajectory. RAST peak power responses showed marked divergence: several CG participants experienced substantial decrements (>200 W). In contrast, intervention groups displayed preservation or modest improvements, though inter-individual variability remained pronounced across all conditions.

## 4. Discussion

The study found that both supplementation timings produced positive effects on recovery and performance outcomes compared with the CG. Specifically, pre-sleep ingestion of casein led to greater improvements in CMJ performance and mean anaerobic power. In contrast, post-exercise ingestion of casein was more effective at enhancing peak power and fatigue resistance. These results suggest that the timing of casein supplementation may differentially influence the recovery process, thereby playing a determining role in subsequent performance outcomes.

Abbott et al. [25] reported that pre-sleep casein ingestion accelerated post-match recovery in soccer players, resulting in significant improvements in CMJ performance and the reactive strength index (RSI) compared with a placebo group. Similarly, Sadeghi et al. [26] demonstrated that combining pre-sleep casein and probiotic supplementation significantly improved anaerobic power output and lower-limb strength among soccer players, resulting in higher peak power values and lower fatigue index scores. Across different sample populations, studies have consistently shown that pre-sleep casein intake benefits recovery and muscle anabolism. Res et al. [10] and Snijders et al. [14] found that consuming casein before sleep after resistance exercise elevated overnight myofibrillar protein synthesis rates, thereby accelerating muscle repair and maintaining an anabolic environment. Likewise, Holwerda et al. [62] reported that pre-sleep casein ingestion in older adults enhanced not only muscle protein synthesis but also connective tissue protein synthesis. Furthermore, Trommelen et al. [63] observed that pre-sleep protein ingestion following endurance exercise promoted a multidimensional recovery response by increasing both myofibrillar and mitochondrial protein synthesis rates. Collectively, these findings are consistent with the present study’s results, indicating that pre-sleep casein ingestion supports recovery and positively influences performance outcomes.

Physiologically, due to its slow digestion rate, casein protein supports muscle protein synthesis and enhances post-exercise recovery by enabling a gradual and sustained release of amino acids into the bloodstream throughout the night when ingested before sleep. This continuous amino acid availability complements the balanced distribution of protein intake during the day, thereby contributing to the maintenance of muscle adaptation and overall protein balance [10,64].

However, some studies in the literature have reported that pre-sleep casein ingestion has no significant or only limited effects on recovery and performance outcomes [65,66,67]. The discrepancies between these findings and the results of the present study may be attributed to various physiological and methodological factors, including differences in the timing of casein administration, the dosage used, the scheduling and intensity of exercise protocols, the extent of exercise-induced muscle damage, and the lack of dietary standardization across experimental conditions.

Casein, due to its high leucine content, slow metabolic rate, and prolonged digestion, is also recommended for post-exercise consumption. These properties enable it to enhance muscle protein synthesis after ingestion, thereby improving athletic performance. A study by Poulios et al. [22] found that post-exercise protein intake accelerated recovery in soccer players. Specifically, the ingestion of milk protein (80% casein and 20% whey) immediately after competition was found to facilitate recovery in performance parameters, including sprint ability, vertical jump height, and knee extensor strength. These findings suggest that post-exercise protein intake may help maintain muscle function, reduce fatigue levels, and accelerate recovery during periods of intense match congestion in soccer players. Consistent with these results, the present study observed that post-exercise consumption of casein positively influenced peak power and fatigue resistance, supporting its beneficial role in post-exercise recovery.

Tipton et al. [13] reported that ingestion of both casein and whey protein immediately after resistance exercise significantly improved the net muscle protein balance. Similarly, Imanian et al. [68] found that combined probiotic and casein supplementation enhanced exercise performance in male soccer players, particularly by improving endurance duration and ventilatory threshold, suggesting that casein supplementation may support aerobic capacity. Consistent with these findings, Mori [69] demonstrated that a single post-exercise ingestion of a protein–carbohydrate mixture acutely improved nitrogen balance, with early intake eliciting a stronger anabolic response compared with delayed ingestion. This finding aligns with the present study, which observed short-term improvements in performance parameters following post-exercise intake of casein. In another study, Joy et al. [70] reported that consuming casein protein either immediately after exercise or before sleep produced similar gains in muscle mass and strength. This result suggests that total protein intake and consistent resistance training may be more critical for muscle adaptations than the specific timing of casein consumption. Likewise, Babault et al. [71] found that a 10-week resistance training program supplemented with casein increased muscle strength and reduced post-exercise muscle fatigue. This supports the notion that casein’s slow digestion provides a prolonged amino acid supply, thereby facilitating recovery. Furthermore, Antonio et al. [72] reported that consuming casein protein in the morning or before sleep produced comparable effects on body composition and performance, supporting the view that overall protein intake, rather than timing, plays a more decisive role in training adaptations.

It is well established that dietary protein intake stimulates muscle protein synthesis, thereby supporting skeletal muscle remodeling following exercise [16]. When combined with resistance exercise, protein intake contributes not only to increases in muscle mass but also to improvements in muscle force-generating capacity [73]. The significant and positive differences observed in anaerobic power output and jump height in the present study may therefore be attributed to this underlying physiological mechanism.

According to the findings of the present study, the timing of casein supplementation may be adjusted based on the specific performance goals of different athlete groups. The demands of training and the required recovery rate appear to be key determinants of optimal timing. During the recovery phase, casein intake supports the repair of damaged muscle fibers. At the same time, nighttime consumption ensures a continuous supply of amino acids, given its slow digestion rate, thereby sustaining muscle protein synthesis throughout the night [10,23,24]. Pre-sleep ingestion of casein may be particularly beneficial for athletes engaged in sports that require explosive strength. In contrast, post-exercise consumption may be more appropriate for those aiming to enhance anaerobic power output and reduce fatigue index. However, the absence of a statistically significant difference in supplementation timing in this study suggests that casein ingestion at either time point can provide meaningful benefits for athletic performance.

### Study Limitations

The present study is among the few studies examining the acute effects of timing of casein supplementation on recovery and anaerobic performance; however, several methodological limitations should be acknowledged. First, the study focused exclusively on short-term responses, thereby precluding conclusions regarding long-term physiological or performance adaptations associated with different casein timing strategies. Second, although the study design aimed to compare pre-sleep and post-exercise ingestion, the absence of a placebo control group may have introduced expectancy-related effects. This limitation arose from the methodological challenge of administering a placebo at two distinct time points without compromising the study’s primary objective. Third, although participants provided dietary records, daily nutritional intake—beyond standardized protein dosing—was not fully controlled, and self-reporting may introduce measurement variability.

Also, the present study employed a fixed 30 g casein dose rather than body-weight-adjusted supplementation (e.g., 0.4 g/kg). This approach was selected based on evidence that absolute protein doses of 20–40 g maximize muscle protein synthesis rates in young adults [36], with doses exceeding 0.4 g/kg providing minimal additional anabolic benefit [18]. Given the narrow body mass range of participants (70.79 ± 5.92 kg), the fixed dose corresponded to 0.38–0.46 g/kg, falling within the recommended optimal range and minimizing inter-individual dosing variability that could confound timing comparisons.

Another limitation is the lack of biochemical markers and physiological measures, such as muscle protein synthesis rates, inflammatory responses, hormonal fluctuations, and oxidative stress indicators, which would have provided greater mechanistic insight. Similarly, sleep duration and quality, which are particularly relevant for pre-sleep supplementation strategies, were not objectively monitored. The relatively small sample size (*n* = 24) may also limit the statistical power of the findings. Furthermore, although players from different positions participated, positional differences in physical demands were not fully controlled or analyzed, which could have influenced variability in recovery and anaerobic performance outcomes. Finally, the study used only acute performance tests (CMJ, IAT, RAST), which, while reliable, do not capture technical–tactical performance or match-play outcomes that recovery interventions may influence.

For athletes and practitioners, both PECIG and PSCIG represent effective strategies for supporting short-term recovery and anaerobic performance. PSCIG may be particularly beneficial for athletes seeking to enhance explosive strength, as reflected in improvements in CMJ performance and mean anaerobic power. Conversely, PECIG may be preferable during training phases that prioritize peak power output and fatigue resistance, offering practical value for athletes engaged in repeated high-intensity sessions. Since neither timing strategy showed superiority overall, casein supplementation can be tailored to individual goals, training schedules, and athlete preferences. Incorporating casein intake during periods of congested match play or intensive training may help maintain performance and mitigate recovery-related decrements, especially when combined with comprehensive nutrition and recovery protocols

## 5. Conclusions

The present study demonstrated that casein supplementation—whether consumed immediately after exercise (PECIG) or before sleep (PSCIG)—produced beneficial short-term effects on recovery and anaerobic performance following high-intensity resistance exercise in soccer players. PSCIG showed greater improvements in countermovement jump height and mean anaerobic power, whereas PECIG showed stronger enhancements in peak power output and fatigue resistance. Although each timing condition favored different performance outcomes, no significant differences were observed between PECIG and PSCIG, indicating that both strategies effectively support recovery and performance. These findings suggest that athletes may flexibly choose the timing of casein ingestion based on their specific performance goals, recovery needs, and daily schedules. Future research should examine the long-term adaptations associated with different casein timing strategies across diverse training modalities and athletic populations, with particular emphasis on mechanisms underlying muscle protein synthesis, hormonal regulation, and recovery biomarkers.

## Figures and Tables

**Table 1:** Mean daily energy and macronutrient intake of participants according to groups.

Groups	Energy (kcal)	CHO (g)	%	Protein (g)	%	Fat (g)	%
CG	3628 ± 175	512 ± 14	56.5 ± 1.2	138 ± 10	14.9 ± 0.1	114 ± 9	28.2 ± 0.8
PECIG	3696 ± 235	520 ± 21	56.3 ± 1.3	142 ± 12	15.3 ± 0.3	116.5 ± 11	28.3 ± 1
PSCIG	3682 ± 112	518 ± 8	56.2 ± 0.8	140.5 ± 5.5	15.2 ± 0.1	116.5 ± 6.5	28.4 ± 0.7

CG = Control Group; PECIG = Post-Exercise Casein Ingestion Group; PSCIG = Pre-Sleep Casein Ingestion Group.

**Table 2:** Demographic and anthropometric characteristics by group.

Variable	CG (*n* = 8) Mean ± SD	CG Range	PECIG (*n* = 8) Mean ± SD	PECIG Range	PSCIG (*n* = 8) Mean ± SD	PSCIG Range
Age (years)	21.38 ± 2.00	19.0–24.0	20.13 ± 1.83	18.0–24.0	20.38 ± 1.49	19.0–24.0
Body Mass (kg)	67.13 ± 4.34	57.0–71.0	73.63 ± 6.26	65.0–85.0	71.63 ± 4.55	67.0–80.0
Height (cm)	172.38 ± 6.40	160.0–180.0	173.38 ± 2.45	170.0–177.0	174.75 ± 4.87	166.0–180.0
BMI (kg/m^2^)	22.61 ± 1.17	20.7–24.9	24.50 ± 2.12	21.7–28.7	23.85 ± 2.04	21.9–27.9
Body Fat (%)	13.49 ± 2.70	10.1–17.8	14.75 ± 2.97	8.7–19.4	16.79 ± 7.66	5.5–33.8

CG = Control Group; PECIG = Post-Exercise Casein Ingestion Group; PSCIG = Pre-Sleep Casein Ingestion Group.

**Table 3:** Performance outcomes: pre-intervention, post-intervention, and change scores.

Outcome	Group	Pre-Intervention Mean ± SD	Post-Intervention Mean ± SD	Change Score Mean ± SD
CMJ (cm)	CG	30.78 ± 4.47	28.49 ± 4.08	−2.29 ± 0.84
	PECIG	33.36 ± 5.79	31.70 ± 5.73	−1.66 ± 1.34
	PSCIG	38.59 ± 4.91	37.75 ± 5.27	−0.84 ± 0.55
IAT Time (s)	CG	17.55 ± 0.82	16.92 ± 0.62	−0.63 ± 0.51
	PECIG	17.34 ± 0.92	17.00 ± 1.01	−0.34 ± 1.03
	PSCIG	16.87 ± 0.95	16.90 ± 0.65	0.03 ± 0.66
RAST Peak Power (W)	CG	663.61 ± 95.78	471.90 ± 139.77	−191.71 ± 103.14
	PECIG	619.67 ± 154.21	633.75 ± 124.89	14.08 ± 144.37
	PSCIG	682.87 ± 159.44	647.02 ± 129.22	−35.84 ± 199.63
RAST Mean Power (W)	CG	513.83 ± 65.18	382.66 ± 75.83	−131.18 ± 57.91
	PECIG	468.60 ± 117.79	465.86 ± 103.89	−2.74 ± 134.62
	PSCIG	545.70 ± 106.64	539.67 ± 91.53	−6.03 ± 98.24
RAST Minimum Power (W)	CG	382.99 ± 62.09	289.92 ± 67.34	−93.07 ± 50.91
	PECIG	344.04 ± 93.00	334.61 ± 60.49	−9.43 ± 123.39
	PSCIG	408.94 ± 108.57	397.77 ± 123.55	−11.17 ± 107.57
RAST Fatigue Index (W/s)	CG	8.45 ± 3.17	5.00 ± 3.48	−3.45 ± 3.37
	PECIG	7.97 ± 3.16	8.64 ± 3.29	0.67 ± 3.00
	PSCIG	8.89 ± 4.68	8.04 ± 4.67	−0.85 ± 7.06

RAST = Running-based Anaerobic Sprint Test; CG = Control Group; PECIG = Post-Exercise Casein Ingestion Group; PSCIG = Pre-Sleep Casein Ingestion Group; CMJ = Countermovement Jump Height; IAT = Illinois Agility Test.

**Table 4:** Comprehensive analysis of performance outcomes: within-group changes and between-group comparisons.

Performance Outcome	Within-Group Pre-Post Changes				Between-Group Comparisons of Change Scores					
	Group	Mean Change (95% CI)	SRM	t_7_, *p*-Value	Comparison	Mean Difference (95% CI)	ES (d)	t_14_, *p*-Value	Statistical Inference	Clinical Magnitude
CMJ (cm)	CG	−2.29 (−2.99, −1.59)	−2.713	−7.673, <0.001 ***	PSCIG vs. CG	1.45 (0.75, 2.15)	2.037	4.074, <0.001 ^†^	Significant	Very Large
	PECIG	−1.66 (−2.78, −0.54)	−1.239	−3.505, 0.001 **	PECIG vs. CG	0.62 (−0.48, 1.73)	0.558	1.116, 0.265	Non-significant	Medium
	PSCIG	−0.84 (−1.30, −0.38)	−1.523	−4.308, <0.001 ***	PSCIG vs. PECIG	0.83 (−0.22, 1.87)	0.805	1.609, 0.108	Non-significant	Large
IAT Time (s)	CG	−0.63 (−1.06, −0.20)	−1.234	−3.492, 0.001 **	PSCIG vs. CG	0.66 (0.07, 1.25)	1.118	2.235, 0.025 ^‡^	Trend	Large
	PECIG	−0.34 (−1.20, 0.52)	−0.330	−0.934, 0.350	PECIG vs. CG	0.29 (−0.49, 1.07)	0.351	0.703, 0.482	Non-significant	Small-Medium
	PSCIG	3/1/2000	0.047	0.134, 0.894	PSCIG vs. PECIG	0.37 (−0.54, 1.28)	0.429	0.859, 0.390	Non-significant	Small-Medium
RAST Peak Power (W)	CG	−191.71 (−277.26, −106.16)	−1.859	−5.257, <0.001 ***	PSCIG vs. CG	155.87 (−1.35, 313.09)	0.981	1.962, 0.050 ^‡^	Trend	Large
	PECIG	14.08 (−106.06, 134.22)	0.098	0.276, 0.783	PECIG vs. CG	205.79 (79.42, 332.16)	1.64	3.281, 0.001†	Significant	Very Large
	PSCIG	−35.84 (−202.16, 130.48)	−0.180	−0.508, 0.612	PSCIG vs. PECIG	−49.92 (−223.15, 123.31)	−0.287	−0.573, 0.567	Non-significant	Small
RAST Mean Power (W)	CG	−131.18 (−179.67, −82.69)	−2.265	−6.406, <0.001 ***	PSCIG vs. CG	125.14 (42.86, 207.42)	1.552	3.104, 0.002 ^†^	Significant	Very Large
	PECIG	−2.74 (−115.06, 109.58)	−0.020	−0.058, 0.954	PECIG vs. CG	128.43 (24.38, 232.48)	1.239	2.479, 0.013 ^†^	Significant	Large
	PSCIG	−6.03 (−88.26, 76.20)	−0.061	−0.174, 0.862	PSCIG vs. PECIG	−3.29 (−119.85, 113.27)	−0.028	−0.056, 0.956	Non-significant	Negligible
RAST Minimum Power (W)	CG	−93.07 (−137.21, −48.93)	−1.828	−5.171, <0.001 ***	PSCIG vs. CG	81.90 (−1.55, 165.35)	0.973	1.947, 0.052 ^‡^	Trend	Large
	PECIG	−9.43 (−113.07, 94.21)	−0.076	−0.216, 0.829	PECIG vs. CG	83.65 (−5.36, 172.66)	0.886	1.773, 0.076 ^‡^	Trend	Large
	PSCIG	−11.17 (−101.67, 79.33)	−0.104	−0.294, 0.769	PSCIG vs. PECIG	−1.75 (−115.21, 111.71)	−0.015	−0.030, 0.976	Non-significant	Negligible
RAST Fatigue Index (W/s)	CG	−3.45 (−6.27, −0.63)	−1.024	−2.896, 0.004 **	PSCIG vs. CG	2.59 (−2.05, 7.23)	0.469	0.937, 0.349	Non-significant	Small-Medium
	PECIG	0.67 (−1.82, 3.16)	0.223	0.631, 0.528	PECIG vs. CG	4.12 (0.87, 7.37)	1.291	2.582, 0.010 ^†^	Significant	Large
	PSCIG	−0.85 (−6.72, 5.02)	−0.121	−0.342, 0.732	PSCIG vs. PECIG	−1.52 (−6.93, 3.89)	−0.281	−0.561, 0.575	Non-significant	Small

**Notes:** Within-group analyses employed paired-sample *t*-tests with Bonferroni-Holm sequential correction for multiple comparisons. Between-group comparisons utilized independent-samples t-tests on change scores with family-wise error rate control (αadjusted = 0.0167). Effect size interpretations follow Cohen’s conventional criteria: negligible (|d| < 0.2), small (0.2 ≤ |d| < 0.5), medium (0.5 ≤ |d| < 0.8), large (0.8 ≤ |d| < 1.2), very large (|d| ≥ 1.2). The Standardized Response Mean (SRM) quantifies the magnitude of within-group change relative to response variability. Clinical magnitude assessments incorporate both statistical significance and practical relevance thresholds. CG = Control Group; PECIG = Post-Exercise Casein Ingestion Group; PSCIG = Pre-Sleep Casein Ingestion Group; RAST = Running-based Anaerobic Sprint Test; CMJ = Countermovement Jump Height; IAT = Illinois Agility Test. ***: *p* < 0.001 (highly significant). **: *p* < 0.01 (very significant). ^†^: *p* < 0.0167 (Bonferroni-corrected significance). ^‡^: 0.05 ≤ *p* < 0.10 (statistical trend).

## Data Availability

The original contributions presented in this study are included in the article. Further inquiries can be directed to the corresponding authors.
